# Complete Genome Sequence of Rhodopseudomonas palustris CGA0092 and Corrections to the R. palustris CGA009 Genome Sequence

**DOI:** 10.1128/mra.01285-22

**Published:** 2023-04-20

**Authors:** Brittany E. Mazny, Olivia F. Sheff, Breah LaSarre, Anastasia McKinlay, James B. McKinlay

**Affiliations:** a Department of Biology, Indiana University, Bloomington, Indiana, USA; University of Delaware College of Engineering

## Abstract

Rhodopseudomonas palustris CGA009 is a versatile model purple nonsulfur bacterium used for both fundamental and applied research. Here, we present a new genome sequence for the derivative strain CGA0092. We further present an improved CGA009 genome assembly that differs from the original CGA009 sequence at three positions.

## ANNOUNCEMENT

Some studies of Rhodopseudomonas palustris CGA009, a model purple nonsulfur bacterium (1), have unknowingly used a variant, here designated CGA0092, that produces less pigment under aerobic conditions. Each strain was obtained from separate 25% glycerol stocks stored at −80°C. Studies with author J. B. McKinlay, except for one study (2), used CGA0092. Strains CGA676 and CGA679 (3–6) were also derived from CGA0092. Here, CGA0092 was sequenced to determine how it differs from the published CGA009 sequence (1).

CGA009 and CGA0092 were grown in anaerobic minimal medium (7) with 10 mM succinate in constant light. Genomic DNA was extracted from 1 mL of the culture using a Qiagen DNeasy blood and tissue kit (catalog number 69504). CGA009 DNA was used for Sanger sequencing (Table 1). Separate CGA0092 DNA batches were processed for Nanopore and Illumina sequencing. The CGA0092 DNA quality was assessed using an Agilent 4200 TapeStation and quantified using a Qubit double-stranded DNA (dsDNA) high-sensitivity (HS) assay kit (catalog number Q32854; Invitrogen) at Indiana University’s Center for Genomics and Bioinformatics. Nanopore libraries were prepared using the ONT rapid sequencing kit (catalog number SQK-RAD004) and were sequenced using a GridION instrument with R9.4.1 flow cells (catalog number FLO-MIN106D) at Indiana University. The following steps were performed at the Microbial Genome Sequencing Center. Libraries were prepared using the Illumina DNA prep kit (catalog number 20018705) with IDT 10-bp unique dual indexes (UDI); the kit included tagmentation that selects for ~280-bp fragments. The libraries were quantified using a Qubit dsDNA HS assay kit before pooling and sequencing on an Illumina NextSeq 2000 instrument, producing 2 × 151-bp reads. Demultiplexing, quality control, and adapter trimming were performed using bcl2fastq v2.20.0.422.

**TABLE 1:** CGA009 sequence errors and the CGA0092 variation

Gene and ORF^*a*^ direction	CGA009 position	Corrections to CGA009 (top strand)	CGA0092 variation	Primers used for Sanger sequencing verification (5′–3′)
*amtB2* →, *RPA0276* →	304135	+G		AmtB2F1 (gtcggcgactacgacatgac)
				AmtB2R1 (gcagtatagcaggcttcggg)
*ppsR2* →	1705143		T → C; C439R	ppsR2F1 (gatcctcgctgcgcaagc)
				ppsR2R1 (ctcgctgacgaccagaagc)
*RPA1790* →	2007023	+A		1790F1 (ggttgcggtgacgatgagc)
				1790R1 (ctcggtgctgctggtggag)
*RPA2040* ←	2297598	ΔG		2040F1 (cgaaagtcttcgccgagcg)
				2040R1 (cacgctgctgcggttgac)

a ORF, open reading frame.

Nanopore sequencing generated 108,118 reads (688,831,264 bp), with an average read length of 6,371 bp. Illumina sequencing generated 3,307,354 reads (822,880,685 bp), with an average read length of 124 bp. Nanopore base calling was performed using Guppy v5.1.13 (8). Trimmomatic v0.36 (9) was used to process both the Illumina (parameters: CROP:140, HEADCROP:15, SLIDINGWINDOW:30:30, MINLEN:100) and Nanopore (parameters: MINLEN:5000) reads. Long-read assembly was performed using Unicycler v0.4.7 (10) in conservative mode; the assembly was polished with the Illumina reads using POLCA (11) and then Polypolish (12), each with default parameters, and assessed using QUAST (13). The assembled genome was compared to the CGA009 sequence (GenBank accession number GCF_000195775.1) using Snippy (14) (parameters, cpus8, contigs). Differing sequences were scrutinized by Sanger sequencing (Eurofins) of the PCR products from both strains (Table 1).

Consistent with CGA009 (1), the CGA0092 assembly comprised one chromosome (5.46 Mb) and one plasmid (8.43 kb). Three CGA009 sequence errors were confirmed (Table 1). CGA009 and CGA0092 differ only by a single nucleotide polymorphism in *ppsR2*, which encodes an O_2_-responsive transcriptional repressor of photosynthesis genes. A cysteine at position 439 (CGA009) results in constitutive pigmentation (15), thus explaining the pigmentation phenotypes and suggesting that CGA0092 is ancestral to CGA009.

### Data availability.

The raw sequencing reads were deposited at NCBI under SRA accession numbers SRX18311788 (Illumina) and SRX18311789 (Nanopore). The BioProject accession number is PRJNA902690. The BioSample accession number is SAMN31769028. The GenBank accession numbers for CGA0092 are CP113083 (chromosome) and CP113084 (plasmid). The updated CGA009 chromosome accession number is CP116810.

## References

[1] Larimer FW, Chain P, Hauser L, Lamerdin J, Malfatti S, Do L, Land ML, Pelletier DA, Beatty JT, Lang AS, Tabita FR, Gibson JL, Hanson TE, Bobst C, Torres JL, Peres C, Harrison FH, Gibson J, Harwood CS. 2004. Complete genome sequence of the metabolically versatile photosynthetic bacterium Rhodopseudomonas palustris. Nat Biotechnol 22:55–61. doi:10.1038/nbt923. 14704707

[2] Huang JJ, Heiniger EK, McKinlay JB, Harwood CS. 2010. Production of hydrogen gas from light and the inorganic electron donor thiosulfate by Rhodopseudomonas palustris. Appl Environ Microbiol 76:7717–7722. doi:10.1128/AEM.01143-10. 20889777 PMC2988585

[3] Heiniger EK, Oda Y, Samanta SK, Harwood CS. 2012. How posttranslational modification of nitrogenase is circumvented in Rhodopseudomonas palustris strains that produce hydrogen gas constitutively. Appl Environ Microbiol 78:1023–1032. doi:10.1128/AEM.07254-11. 22179236 PMC3273035

[4] Fixen KR, Zheng Y, Harris DF, Shaw S, Yang ZY, Dean DR, Seefeldt LC, Harwood CS. 2016. Light-driven carbon dioxide reduction to methane by nitrogenase in a photosynthetic bacterium. Proc Natl Acad Sci USA 113:10163–10167. doi:10.1073/pnas.1611043113. 27551090 PMC5018805

[5] Corneli E, Adessi A, Dragoni F, Ragaglini G, Bonari E, De Philippis R. 2016. Agroindustrial residues and energy crops for the production of hydrogen and poly-beta-hydroxybutyrate via photofermentation. Bioresour Technol 216:941–947. doi:10.1016/j.biortech.2016.06.046. 27341463

[6] Corneli E, Adessi A, Olguin EJ, Ragaglini G, Garcia-Lopez DA, De Philippis R. 2017. Biotransformation of water lettuce (Pistia stratiotes) to biohydrogen by Rhodopseudomonas palustris. J Appl Microbiol 123:1438–1446. doi:10.1111/jam.13599. 28972701

[7] Kim M-K, Harwood CS. 1991. Regulation of benzoate-CoA ligase in Rhodopseudomonas palustris. FEMS Microbiol Lett 83:199–203. doi:10.1111/j.1574-6968.1991.tb04440.x-i1.

[8] Wick RR, Judd LM, Holt KE. 2019. Performance of neural network basecalling tools for Oxford Nanopore sequencing. Genome Biol 20:129. doi:10.1186/s13059-019-1727-y. 31234903 PMC6591954

[9] Bolger AM, Lohse M, Usadel B. 2014. Trimmomatic: a flexible trimmer for Illumina sequence data. Bioinformatics 30:2114–2120. doi:10.1093/bioinformatics/btu170. 24695404 PMC4103590

[10] Wick RR, Judd LM, Gorrie CL, Holt KE. 2017. Unicycler: resolving bacterial genome assemblies from short and long sequencing reads. PLoS Comput Biol 13:e1005595. doi:10.1371/journal.pcbi.1005595. 28594827 PMC5481147

[11] Zimin AV, Salzberg SL. 2020. The genome polishing tool POLCA makes fast and accurate corrections in genome assemblies. PLoS Comput Biol 16:e1007981. doi:10.1371/journal.pcbi.1007981. 32589667 PMC7347232

[12] Wick RR, Holt KE. 2022. Polypolish: short-read polishing of long-read bacterial genome assemblies. PLoS Comput Biol 18:e1009802. doi:10.1371/journal.pcbi.1009802. 35073327 PMC8812927

[13] Gurevich A, Saveliev V, Vyahhi N, Tesler G. 2013. QUAST: quality assessment tool for genome assemblies. Bioinformatics 29:1072–1075. doi:10.1093/bioinformatics/btt086. 23422339 PMC3624806

[14] Seemann T. 2015. Snippy: fast bacterial variant calling from NGS reads.

[15] Fixen KR, Harwood CS. 2016. A polymorphism in the oxygen-responsive repressor PpsR2 confers a growth advantage to Rhodopseudomonas palustris under low light. Photosynth Res 129:199–204. doi:10.1007/s11120-016-0288-0. 27344652

